# Books and Pamphlets Received

**Published:** 1885-08-20

**Authors:** 


### BOOKS AND PAMPHLETS RECEIVED.

*Urinary and Renal Derangements and Calculous Disorders;* Hints on Diagnosis and Treatment. By Lionel S. Beale, M. D. Philadelphia : P. Blakiston, Son & Co.

The very name of the author should, and we have no doubt will, secure for this admirable manual an unprecedented demand. Its value, as a work for daily reference, cannot be questioned, and may be regarded as a standard guide removing many of the obstacles which the student always, and the practitioner often, meet with, while studying disease in its Protean forms at the bedside. We consider this the most valuable contribution that has been made of late to the diagnosis and treatment of renal troubles, and take pleasure in recommending it to the profession in general and to the student in particular.

*Clinical Studies of Diseases of the Eye*, including those of the Conjunctiva, Cornea, Schlerotie, Iris, and Ciliary body. By Dr. Ferdinand Ritter von Arlt, Professor of Ophthalmology in Viena ; translated by Lyman Ware, M. D., Surgeon to the Illinois Charitable Eye and Ear Infirmary, Ophthalmic Surgeon to the Presbyterian Hospital, and to the Protestant Orphan Asylum, Chicago. Philadelphia : P. Blakiston, Son & Co. 1885.

This work has 325 octavo pages, and is an embodiment of Professor Arlt's clinical-studies during nearly half a century of ophthalmic practice. The distinctive original features of the work are preserved by the translator, who has carefully abstained from adding to the text. He has, however, included an account of the new local anæsthetic, or cocaine, which is an important item discovered since the German edition was issued.

*Kirk's Handbook of Physiology.* By W. Morrant Baker, F. R. C. S., Surgeon to St. Bartholomew's Hospital, and Consulting Surgeon to the Evelina Hospital for Sick Children ; Lecturer on Physiology at St. Bartholomew's Hospital, and late member of the Board of Examiners of the Royal College of Surgeons of England ; and Vincent Dormer Harris, M.D., Demonstrator of Physiology at St. Bartholomew's Hospital, London. Eleventh edition, with nearly 500 illustrations ; in two volumes. New York : William Wood & Co., 56 and 68 LaFayette Place. 1885.

The old and favorite text-book on physiology is here presented in a new and revised edition of two volumes of about 375 pages each. New facts and observations have been incorporated so as to bring the work up to the latest advances, and many new illustrations have been added.

This work has been so long and favorably known that it seems scarcely necessary to make mention of its excellencies as a text-book. We regard it as among the very best works for the student now extant. We like the order and arrangement in which the several subjects are treated, and the plain, condensed and yet comprehensive style of the work ; and, while we think it a mistake to divide the work into two volumes, we nevertheless esteem it highly, and can recommend it as a standard and highly valuable text-book on Physiology.

*The Technology of Bacteria Investigation.* Explicit directions for the study of Bacteria, their culture, mounting, etc , according to the methods employed by the most eminent investigators. By Charles S. Dolley, M. D. Boston : S. E. Cassens & Co. 1885.

A work of 263 pages, containing much interesting and useful information in regard to bacteria as found in the air, water, earth, and in various fluids, and tissues of living organisms. It is a work of rare interest to the investigating student ; useful not only for the facts and observations it contains, but especially so in the instruction it affords in the methods used for the study of bacteria, which are grouped under four heads : 1. Microscopical preparations : 2. Culture experiments ; 3. Vaccination, or innoculation, experiments ; 4. Biological analyses.

*A Manual of Obstetrics.* By Edward L. Partridge, M. D., Professor of Obstetrics, New York Post Graduate Medical School ; Instructor in Obstetrics, College of Physicians and Surgeons, New York ; Visiting Physician to the Maternity Hospital, and to the Nursery and Child's Hospital ; Attending Gynecologist to the New York Hospital, Out Patient department ; Fellow of the New York Obstetrical Society. With illustrations. New York : William Wood & Co. 1885.

This is a small, compact work, which may be carried in the pocket, containing a concise outline of obstetric knowledge, useful to the medical student as a reminder of his teachings, when about to pass his final examination, and to the practitioner as furnishing safe and ready suggestions in times of emergency.

*Minor Surgical Gynecology :* a Treatise of Uterine Diagnosis, and the lesser technicalities of Gynecological Medicine, including general rules for gynecological operations, and the operations for lacerated cervix and perineum, and prolapsus of uterus and vagina, for the use of the advanced student and general practitioner. By Paul F. Munde, M. D., Professor of Gynecology a the New York Polyclinic, and at Dartmouth College ; Gynecologist to Mount Senia Hospital ; Obstetric Surgeon to Maternity ; Vice-President of the American Gynecological Society of New York ; Corresponding Fellow of the Obstetrical Societies of Edinburgh and Philadelphia, and of the Gynecological Society of Boston, etc. Second edition ; revised and enlarged, with 321 illustrations. New York : William Wood & Co., 56 and 58 LaFayette Place. 1885.

After a careful examination of the above work, we hesitate not to pronounce it a very timely and valuable one. The book contains 550 large octavo pages, devoted to making plain and intelligible the manipulations, etc., in the diagnosis and treatment of uterine affections. The author truly remarks that " It is not a detailed account of larger operations which the general practitioner needs; these he can study up for special cases when such occur to him, or he will probably transfer them to some specialist ; but a knowledge of all the minute technicalities of local examination, digital and instrumental, and of the various manipulations and minor operations which he is liable to meet with every day." This information the author has sought to supply in the above work, and we think with as large a degree of success as possible. The book should be in the library of every practitioner of medicine.

*The Ten Laws of Health*, or How Diseases are Produced and Prevented, and Family Guide to Protection against Epidemic Diseases and other dangerous Infections. By J. R. Black, M. D. Philadelphia: J. B. Lippincott Company. 1885. Price, $2.

This is the third and revised edition of the work of which the above is the title page. It is an interesting work of over 400 pages, and the present volume is adapted to the advanced views of the present time, in reference to the germ theory of disease—the author evidently accepting the theory that viable germs are the means by which deadly infections are propagated. The importance of this doctrine in its bearings upon the prevention of disease is clearly and forcibly set forth. The work contains much valuable information, and the views of the author are presented in a manner easily comprehended even by the non-professional reader.

☞ RECEIPTS will appear in our next.

